# The Beneficial Effect of Pollen on Varroa Infested Bees Depends on Its Influence on Behavioral Maturation Genes

**DOI:** 10.3389/finsc.2022.864238

**Published:** 2022-04-27

**Authors:** Davide Frizzera, Allyson M. Ray, Elisa Seffin, Virginia Zanni, Desiderato Annoscia, Christina M. Grozinger, Francesco Nazzi

**Affiliations:** ^1^Department of Agricultural, Food, Environmental and Animal Sciences (DI4A), University of Udine, Udine, Italy; ^2^Molecular, Cellular, and Integrative Biosciences Graduate Program, The Huck Institutes of the Life Sciences, The Pennsylvania State University, University Park, PA, United States

**Keywords:** honey bee, nutrition, pollen, juvenile hormone, behavioral maturation, Vitellogenin

## Abstract

Honey bees collect nectar and pollen to fulfill their nutritional demands. In particular, pollen can influence longevity, the development of hypopharyngeal glands, and immune-competence of bees. Pollen can also mitigate the deleterious effects caused by the parasitic mite *Varroa destructor* and related deformed wing virus (DWV) infections. It has been shown that *V. destructor* accelerates the physiological and behavioral maturation of honey bees by influencing the interaction between two core physiological factors, Vitellogenin and juvenile hormone. In this study, we test the hypothesis that the beneficial effects of pollen on Varroa-infested bees are related to the hormonal control underpinning behavioral maturation. By analyzing the expression of genes associated to behavioral maturation in pollen-fed mite-infested bees, we show that treatment with pollen increases the lifespan of mite-infested bees by reversing the faster maturation induced by the parasite at the gene expression level. As expected, from the different immune-competence of nurse and forager bees, the lifespan extension triggered by pollen is also correlated with a positive influence of antimicrobial peptide gene expression and DWV load, further reinforcing the beneficial effect of pollen. This study lay the groundwork for future analyses of the underlying evolutionary processes and applications to improve bee health.

## Introduction

Honey bees use carbohydrates to obtain energy, proteins for growth and development, lipids for energy reserves, whereas minerals, vitamins and water are needed for optimal survival (1). Honey bees gather these substances by collecting nectar, pollen and water from the environment in quantities that can exceed colony demands. The surplus is stored for periods of dearth and for feeding juvenile stages (2). Nectar is the only source of carbohydrates; it provides energy for metabolic processes but it is also associated with the innate humoral and cellular immune reactions. Nectar can also provide secondary plant metabolites complementing the immune system reducing microbial or pathogen pressure due to their antimicrobial properties (3). Pollen provides proteins, lipids, amino acids, sterols and vitamins required for physiological processes such as growth and immunity (4–6). Indeed, these nutrients make pollen nutrition one of the most important factors influencing bee longevity (7) and a key factor boosting honey bee tolerance against pesticides, pathogens and viruses (8–11). Pollen nutrition also positively affects the development of hypopharyngeal glands (12), the production of antimicrobial peptides (AMPs) (13), the expression of longevity genes (13), and generally increases immune competence (11, 14).

The mite *Varroa destructor* is the most important ecto-parasite of the western honey bee (15). During the reproductive phase, inside the capped brood cells, the mite feeds on the haemolymph obtained from a hole pierced in the pupal abdomen (16). Recent work suggests that Varroa mites also consume materials from the fat body while they are feeding from the pupating bee (17) but further research is needed to determine the extent to which the nutritional needs of the mite are met by the residual fat body vs. the hemolymph. This feeding activity is central to all the detrimental effects caused by the parasite (15), although it is difficult to distinguish between the direct effects of mite parasitization and the indirect ones related to the viruses vectored and facilitated by the mite. In particular, Varroa can transmit and promote the replication of deformed wing virus (DWV) (18, 19), which, due to its ubiquitous distribution (20), represents a constant threat to the survival of honey bee colonies (21).

Varroa can also compromise the normal relationship between nutrition and immunity (22). Indeed, mite parasitized bees have a lower weight at the emergence, lower protein content and elevated free amino acids levels, suggesting that protein synthesis and growth are disrupted by Varroa (23). Varroa also influences the food intake of adult honey bees parasitized at the pupal stage (i.e., parasite induced anorexia), likely due to an interaction with the insulin pathway (24). Varroa can additionally modulate the honey bee's age-dependent behavioral maturation. Worker bees start out as hive bees nursing larvae and subsequently switch to foraging and this transition represents a turning point in the honey bee's life, involving drastic changes in behavior, physiology and immunity, with foragers showing reduced immune-competence as compared to nurses [for a review, see (25)]. The mite accelerates the physiological maturation of honey bees by influencing the relationship between two core physiological factors, Vitellogenin and juvenile hormone (JH) (26–28). Vitellogenin is a yolk precursor protein that is also involved in immunity (29, 30). The protein, encoded by *vg* gene, is particularly abundant in the haemolymph of nurse honey bees (31–33). JH is a hormone with high titers in forager bees and a low concentration in nurses (34, 35). Juvenile hormone esterases (JHEs), play a major role in JH metabolism. In particular, JH esterase, encoded by *jhe*, degrades JH during larval and pupal development and can therefore be used as marker of JH titer (35).

Nurse bees are thus characterized by a high concentration of Vitellogenin and a low JH titer, while foragers show low levels of the protein and high hormone concentration. These two compounds are involved in a double negative feedback loop that regulates forager transition (36, 37). Moreover, the timing of the switch determines the overall lifespan of the worker (25) such that the transition to foraging can be interpreted as the starting point of a count-down to death.

In a previous study, we showed that pollen intake can mitigate the deleterious effects of *V. destructor* and the related virus infections enhancing the lifespan of mite-infested bees under lab conditions (38). In the article we listed a number of possible mechanisms accounting for the observed beneficial effects of pollen on diseased bees, including: increasing the supply of energetic compounds complementary to sugars (i.e., lipids), reinforcing the cuticle and thus preventing water loss, improving defense against pathogens facilitated by Varroa; influencing the hormonal regulation of the honey bee's homeostasis. This latter potential mechanism is particularly interesting for the possible implications for polyethism which is regulated by hormones.

Here we test the hypothesis that pollen can prolong mite-infested bees' lifespan by inverting the accelerated behavioral maturation caused by the parasite. Since Vitellogenin and JH are the key regulators of behavioral maturation in bees (36, 39), we predicted that pollen stimulates the expression of *vg* and *jhe* in mite-infested bees (prediction 1). Moreover, we predicted that the delay of the transition to foraging caused by pollen should stimulate immunity (prediction 2) because the transition to foraging is associated with a reduced immune-competence (29, 40, 41). To this aim, we used *Apidaecin-1* and *Defensin-1* as indicators of immune system activation. Furthermore, given the reduced immune-competence associated with the transition to foraging, we predicted that the abundance of DWV is affected by age and pollen feeding (prediction 3).

To test our hypothesis and the related predictions, we artificially infested honey bee larvae at the pupal stage and fed the eclosing bees with a diet complemented with or without pollen. Then, after confirming the beneficial effect of pollen on the lifespan of mite-infested honey bees, we studied the expression of key genes involved in behavioral maturation and immunity to assess how they are affected by parasitization and how this influence is shaped by the pollen acquired through the diet.

## Materials and Methods

### Effect of Pollen on Varroa-Infested Honey Bees

To confirm that pollen can mitigate the negative effect of mite parasitization and assess the expression of a panel of key genes involved in behavioral maturation and immunity, we reared honey bee larvae inside artificial cells in presence of a Varroa mite or not according to Nazzi and Milani (42). To this aim, we transferred 5th instar larvae into gelatine capsules (6.5 mm Ø; Agar Scientific Ltd.) with one (V+) or no (V–) mites and maintained them in an incubator at 34.5°C, 75% relative humidity (R.H.), dark, for 12 days. At the emergence, Varroa-infested bees (that were separated from the mite) and control bees were transferred into plastic cages (185 × 105 × 85 mm), under standardized environmental conditions (34.5°C, 75% R.H., dark) and fed under two different diet regimes: a sugar diet complemented with pollen (P+) and a sugar diet (P–), supplied *ad libitum*. Sugar was provided as a solution (61% glucose, 39% fructose) with a 20 mL syringe, whereas multifloral pollen (previously maintained at −20°C) was offered to bees in an open petri dish placed on the floor of the cages. Sugar solution and pollen were renewed every 7 days.

In total, we set up four experimental groups (from 54 to 62 bees per group): uninfested bees fed with sugar only (V–P–), uninfested bees fed with sugar and pollen (V–P+), mite-infested bees fed with sugar (V+P–) and mite-infested bees fed with sugar and pollen (V+P+).

Dead bees were counted and removed daily. The experiment was replicated three times.

### Gene Expression

Bees to be used for the molecular analyses were sampled on day 7 and 14 and flash frozen in liquid nitrogen. In those bees, we studied the expression of the following genes: (1) *vg* (Supplementary Table 1), which encodes for Vitellogenin; (2) *jhe* (Supplementary Table 1); (3) *Apidaecin-1* and 4. *Defensin-1* (Supplementary Table 1). We also tested the abundance of DWV (Supplementary Table 1). According to Corona et al. (43) *vg* expression varies across tissues, being the highest in the abdomen where fat body is concentrated; however, the time-dependent pattern of expression is the same in different body parts. For this reason, in this study, which was dedicated to the possible influence of diet and mite infestation on worker bees' behavioral maturation, we investigated gene expression using the whole body of honey bees.

Sampled bees were homogenized using mortar and pestle in liquid nitrogen. Total RNA was extracted from each bee according to the protocol provided with the RNeasy Plus mini kit (Qiagen^®^, Germany). The amount of RNA in each sample was quantified with a NanoDrop^®^ spectrophotometer (ThermoFisher™, US). cDNA was synthesized from 500 ng of RNA per sample, following the manufacturer specifications (PROMEGA, Italy). Additional negative control samples containing no RT enzyme were included. Ten nanogram of cDNA from each sample were analyzed using SYBR^®^ Green dye (Ambion^®^) according to the manufacturer specifications, on a BioRad CFX96 Touch™ Real time PCR Detector. All samples were run in duplicate; when technical replicates differed by more than 0.5 Ct, the analysis was repeated, in duplicate, in another plate. The following thermal cycling profiles were adopted: one cycle at 95°C for 10 min, 40 cycles at 95°C for 15 s and 60°C for 1 min, and one cycle at 68°C for 7 min. Given the high number of samples to be analyzed, an inter-plate calibrator (i.e., a control sample that was run in every analyzed plate) was used. Relative viral load and gene expression were analyzed with the 2^−Δ*ΔCt*^ method (44) using b-actin and GAPDH as housekeeping genes (Supplementary Table 1); those genes were selected on the ground of literature data and a preliminary study aiming at comparing the response of some candidate housekeeping genes. Primers' efficiency was between 95 and 99%. Log Normalized values were analyzed using GLM by means of Minitab 16. In total, five bees per treatment and per sampling point were analyzed. All data and the details of the statistical analyses are reported in Supplementary Data Sheet 1.

## Results

### Effect of Pollen and Varroa on Honey bee Survival

**Figure 1:**
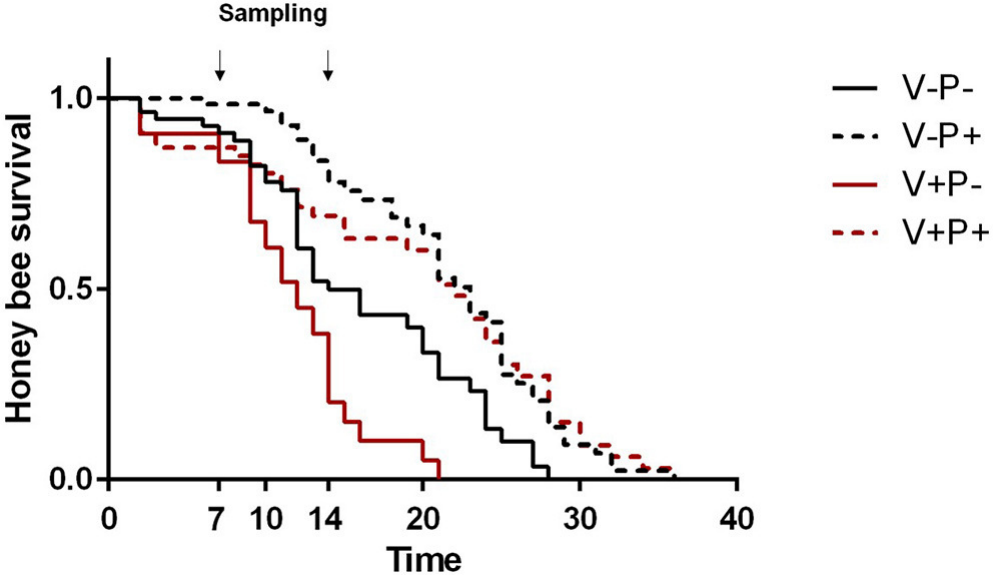
Survival of uninfested (V–) and mite-infested (V+) honey bees fed with pollen (P+) or not (P–). Varroa decreased the survival of honey bees [V–P– vs. V+P-, Log Rank (Chi Square = 10.59, d.f. = 1, *P* = 0.001)]. Pollen increased the survival of both uninfested [V–P– vs. V–P+, Log Rank (Chi Square = 13.31, d.f. = 1, *P* < 0.001)] and mite-infested honey bees [V+P- vs. V+P+, Log Rank (Chi Square = 22.77, d.f. = 1, *P* < 0.001)]. The survival of Varroa-infested bees fed with pollen was similar to that of pollen-fed uninfested bees [V+P+ vs. V–P+, Log Rank (Chi Square = 0.096, d.f. = 1, *P* = 0.757)]. Arrows indicate the timing of sampling for gene expression analysis at day 7 and 14.

As expected, under lab conditions, Varroa significantly negatively impacted honey bee survival [Figure 1; V–P– vs. V+P–, Log Rank (Chi Square = 10.59, d.f. = 1, *P* = 0.001)] while pollen positively influenced the lifespan of healthy honey bees [Figure 1; V–P– vs. V–P+, Log Rank (Chi Square = 13.31, d.f. = 1, *P* < 0.001)]. Also, pollen significantly increased the lifespan of Varroa-infested honey bees [Figure 1; V+P– vs. V+P+, Log Rank (Chi Square = 22.77, d.f. = 1, *P* < 0.001)] such that the survival curve of parasitized bees closely resembled that of uninfested bees [Figure 1; V+P+ vs. V–P+, Log Rank (Chi Square = 0.096, d.f. = 1, *P* = 0.757)].

### Effect of Pollen and Varroa on Genes Involved in Behavioral Maturation

Vitellogenin and juvenile hormone play a fundamental role in lifespan and behavioral maturation. The protein is high in nurses and low in foragers, while JH follows an opposite pattern. Therefore, we studied the expression of *vg*, the gene encoding Vitellogenin, and *jhe*, which encodes JH esterase, in relation to pollen diet, Varroa infestation and sampling time. Since *vg* expression is related to Vitellogenin synthesis while *jhe* expression is involved in JH degradation, *vg* and *jhe* are expected to be both high in nurses and low in foragers.

**Figure 2:**
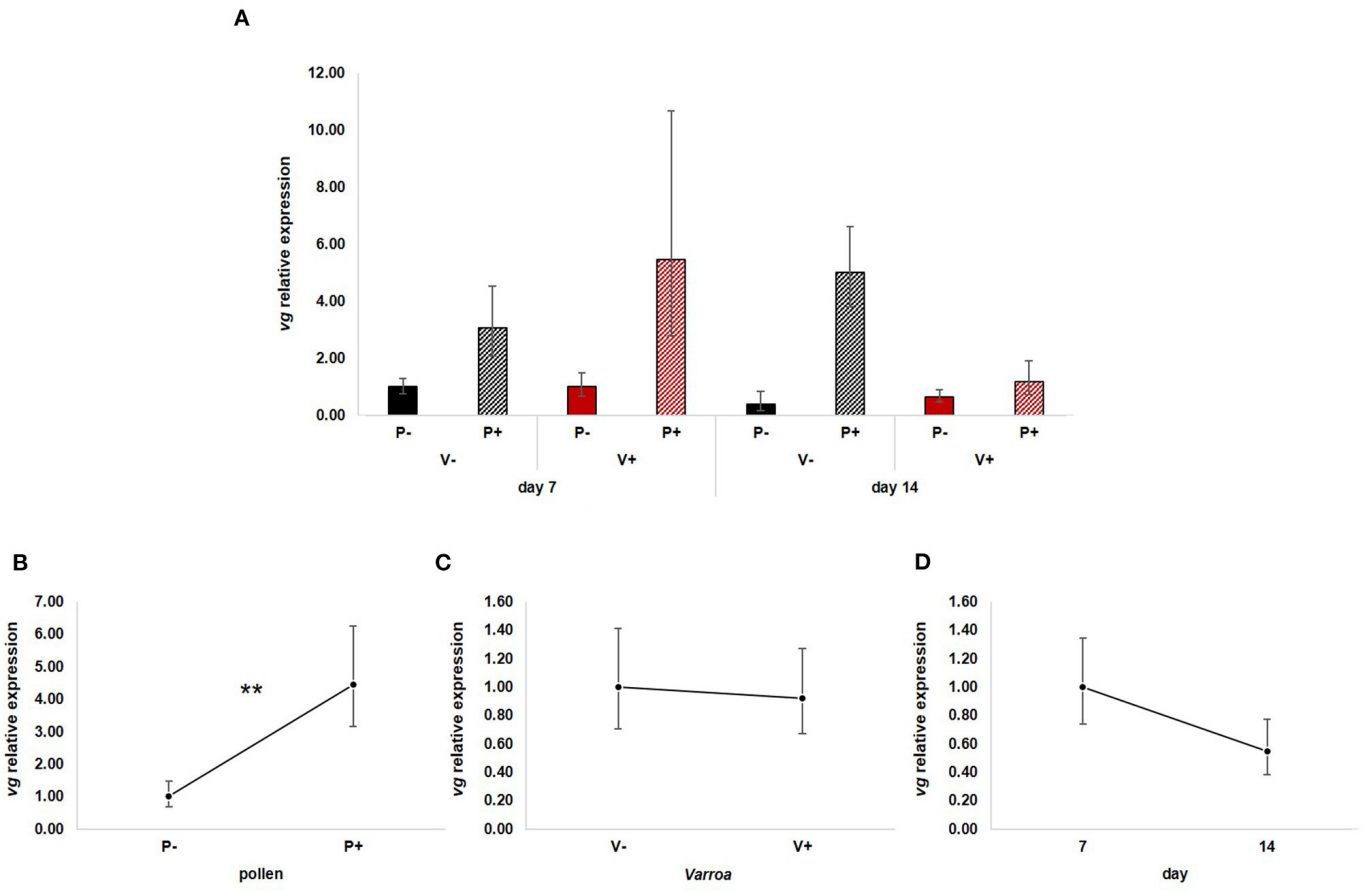
**(A)** *vg* relative expression in the experimental groups. **(B)** Pollen significantly up-regulated *vg* expression [GLM, pollen (*F* = 10.71, d.f. = 1, *P* = 0.003)]. **(C)** Varroa did not have a significant effect on *vg* expression [GLM, Varroa (*F* = 0.01, d.f. = 1, *P* = 0.941)]. **(D)** *vg* expression decreased with time, but the effect was not significant [GLM, Time (*F* = 1.73, d.f. = 1, *P* = 0.197)]. **P < 0.01.

GLM analysis showed that *vg* expression (Figure 2A) is significantly up-regulated by pollen [Figure 2B; GLM, pollen (*F* = 10.71, d.f. = 1, *P* = 0.003)] but not by Varroa [Figure 2C; GLM, Varroa (*F* = 0.01, d.f. = 1, *P* = 0.941)] and time [Figure 2D; GLM, Time (*F* = 1.73, d.f. = 1, *P* = 0.197)]. No significant interactions among the three factors were noted (Supplementary Figures 1A–C).

**Figure 3:**
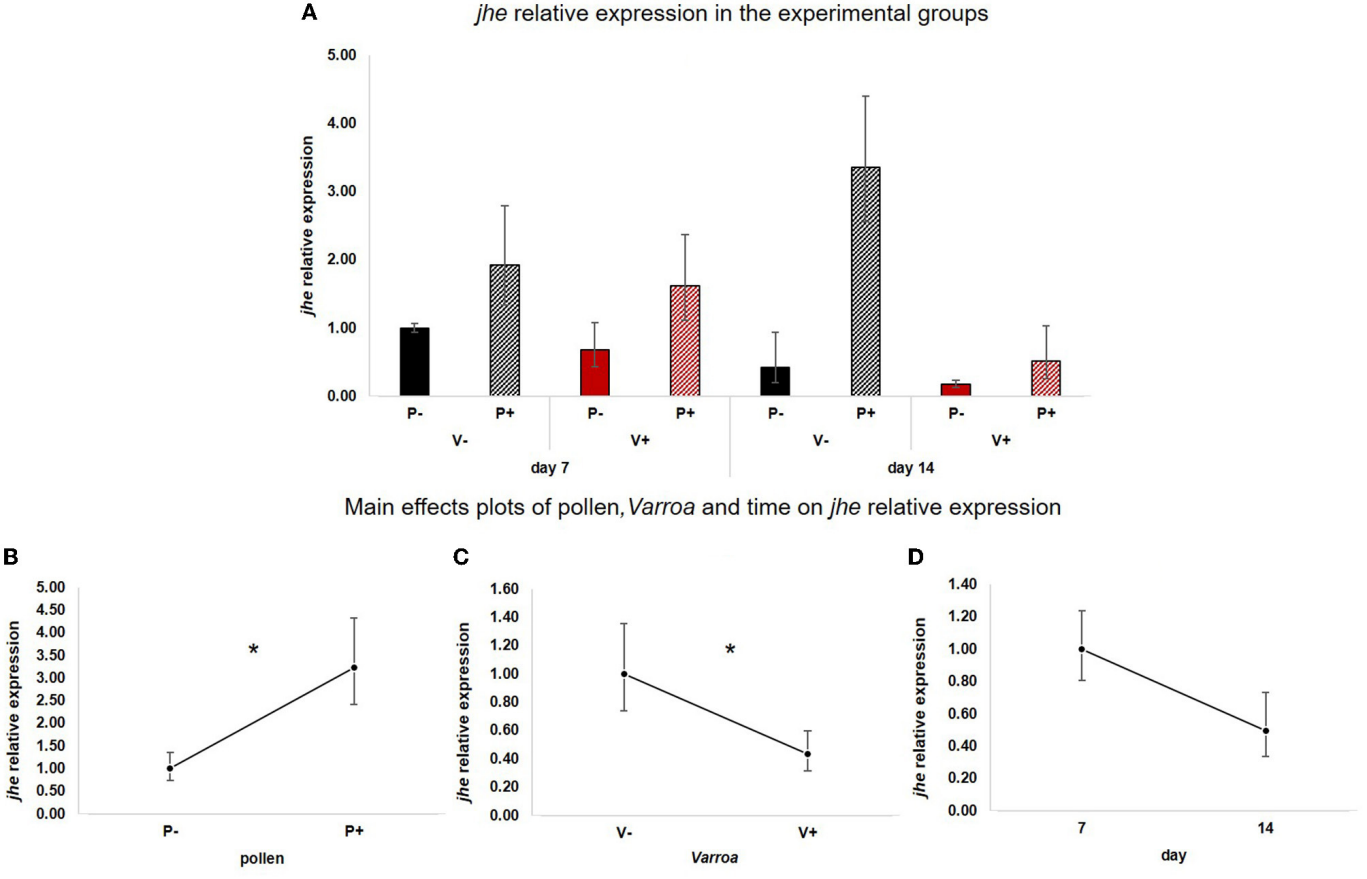
**(A)** *jhe* relative expression in the experimental groups. **(B)** Pollen significantly up-regulated *jhe* [GLM, pollen (*F* = 7.61, d.f. = 1, *P* = 0.01)]. **(C)** Varroa significantly down-regulated *jhe* [GLM, Varroa (*F* = 4.15, d.f. = 1, *P* = 0.047)]. **(D)** *jhe* expression decreased with time, but the effect is not significant [GLM, time (*F* = 1.13, d.f. = 1, *P* = 0.297)]. *P < 0.05.

Likewise, *jhe* (Figure 3A) was positively influenced by pollen [Figure 3B; GLM, pollen (*F* = 7.61, d.f. = 1, *P* = 0.01)] but was also significantly down-regulated by Varroa [Figure 3C; GLM, Varroa (*F* = 4.15, d.f. = 1, *P* = 0.047)]. Time did not significantly affect the expression of this gene [Figure 3D; GLM, Time (*F* = 1.13, d.f. = 1, *P* = 0.297)]. No significant interactions between pollen, Varroa and time were noted (Supplementary Figures 2A–C).

### Effects of Pollen and Varroa on Antimicrobial Peptides

The transition to foraging is associated with a reduced immune-competence, and antimicrobial peptides are key immune effectors. Therefore, we tested if this further indicator of aging is affected by mite infestation and assessed how pollen feeding shapes this interaction.

**Figure 4:**
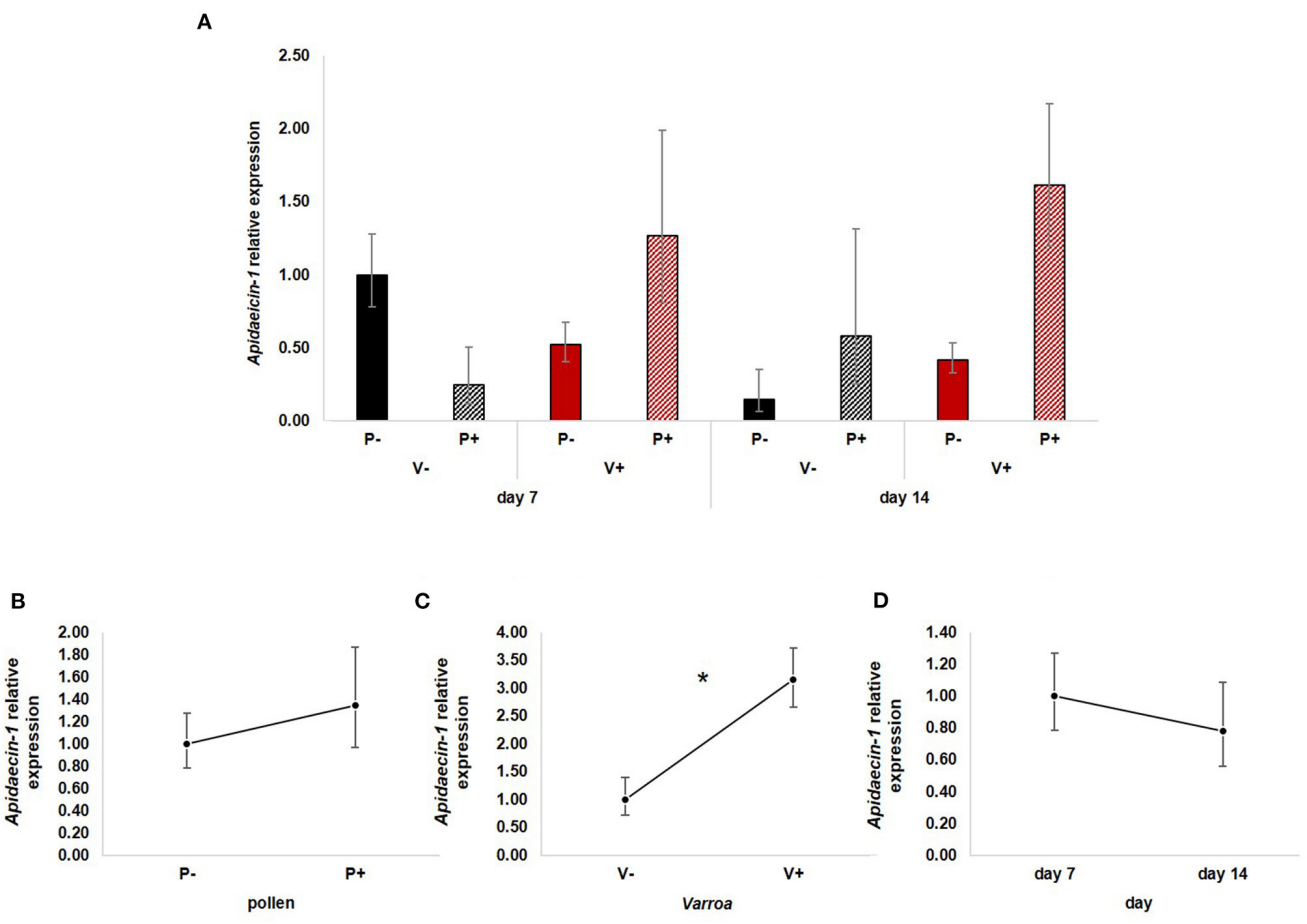
**(A)** *Apidaecin-1* relative expression in the experimental groups. **(B)** Pollen does not have significant effects on *Apidaecin-1* expression [GLM, pollen (*F* = 2.17, d.f. = 1, *P* = 0.148)]. **(C)** Varroa significantly up-regulates *Apidaecin-1* expression [GLM, Varroa (*F* = 6.31, d.f. = 1, *P* = 0.015)]. **(D)** No significant effect of time on *Apidaecin-1* expression [GLM, time (*F* = 0.19, d.f. = 1, *P* = 0.667)]. *P < 0.05.

*Apidaecin-1* expression (Figure 4A) was not statistically influenced by pollen [Figure 4B; GLM, pollen (*F* = 2.17, d.f. = 1, *P* = 0.148)] and time [Figure 4D; GLM, time (*F* = 0.19, d.f. = 1, *P* = 0.667)] while mite infestation activated the expression of this AMP [Figure 4C; GLM, Varroa (*F* = 6.31, d.f. = 1, *P* = 0.015)]. The interaction between Varroa and pollen was close to significance (Supplementary Figure 3A); no other significant interactions were noted (Supplementary Figures 3B,C).

**Figure 5:**
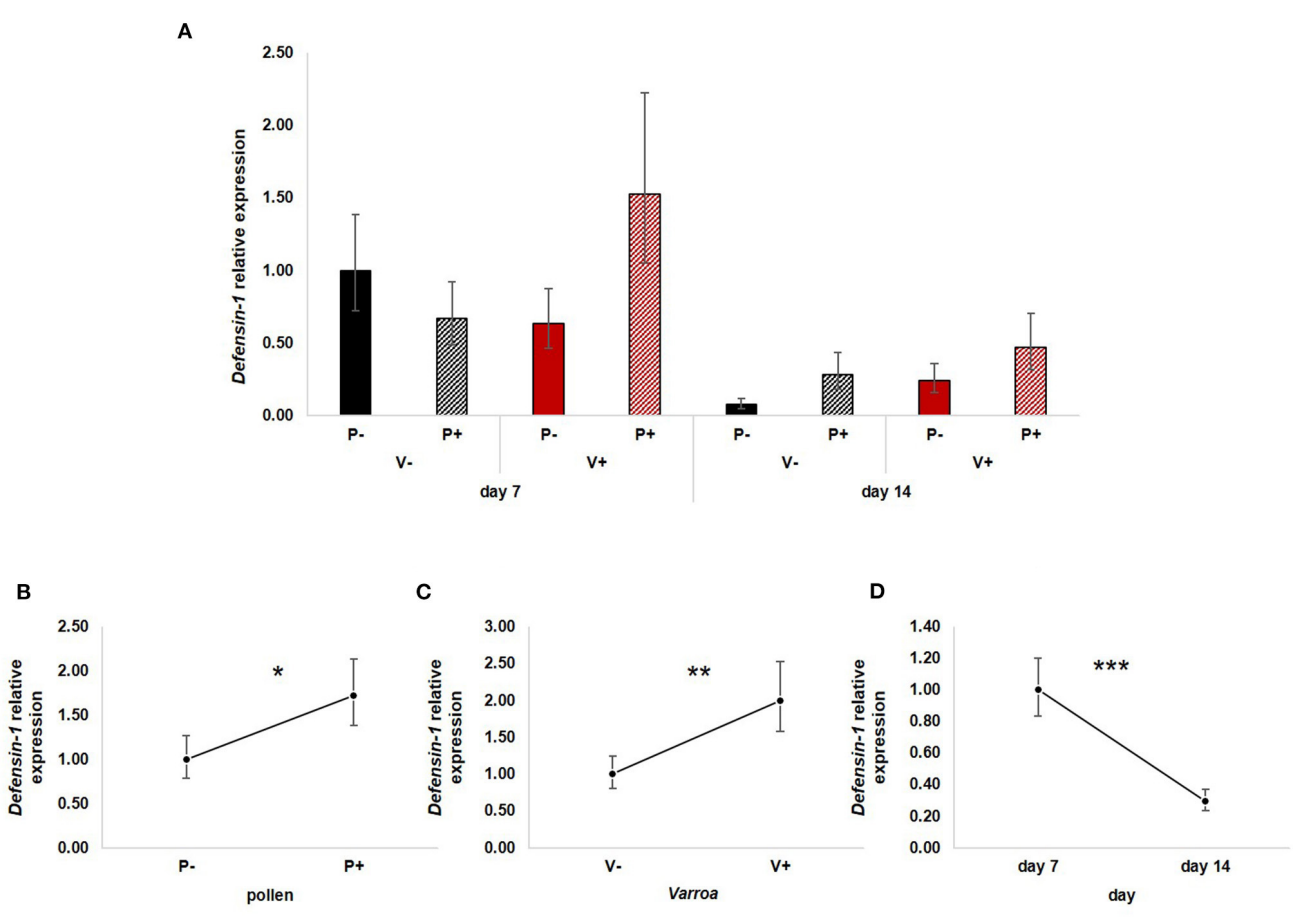
**(A)** *Defensin-1* relative expression in the experimental groups. **(B)** Pollen significantly up-regulated *Defensin-1* expression [GLM, pollen (*F* = 4.22, d.f. = 1, *P* = 0.045)]. **(C)** Varroa significantly increased *Defensin-1* expression [GLM, Varroa (*F* = 7.48, d.f. = 1, *P* = 0.009)]. **(D)** *Defensin-1* expression significantly decreased with time [GLM, time (*F* = 19.54, d.f. = 1, *P* < 0.001)]. *P < 0.05; **P < 0.01; ***P < 0.001.

All three factors—pollen, Varroa and time—had a significant effect on the expression of *Defensin-1* (Figure 5A). In particular, pollen up-regulated *Defensin-1* expression [Figure 5B; GLM, pollen (*F* = 4.22, d.f. = 1, *P* = 0.045)] as well as Varroa [Figure 5C; GLM, Varroa (*F* = 7.48, d.f. = 1, *P* = 0.009)], while the expression decreased with time [Figure 5D; GLM, time (*F* = 19.54, d.f. = 1, *P* < 0.001)]. The interaction between Varroa and pollen was close to significance (Supplementary Figure 4A); no other significant interactions were noted (Supplementary Figures 4B,C).

### Effects of Pollen on Viral Load

**Figure 6:**
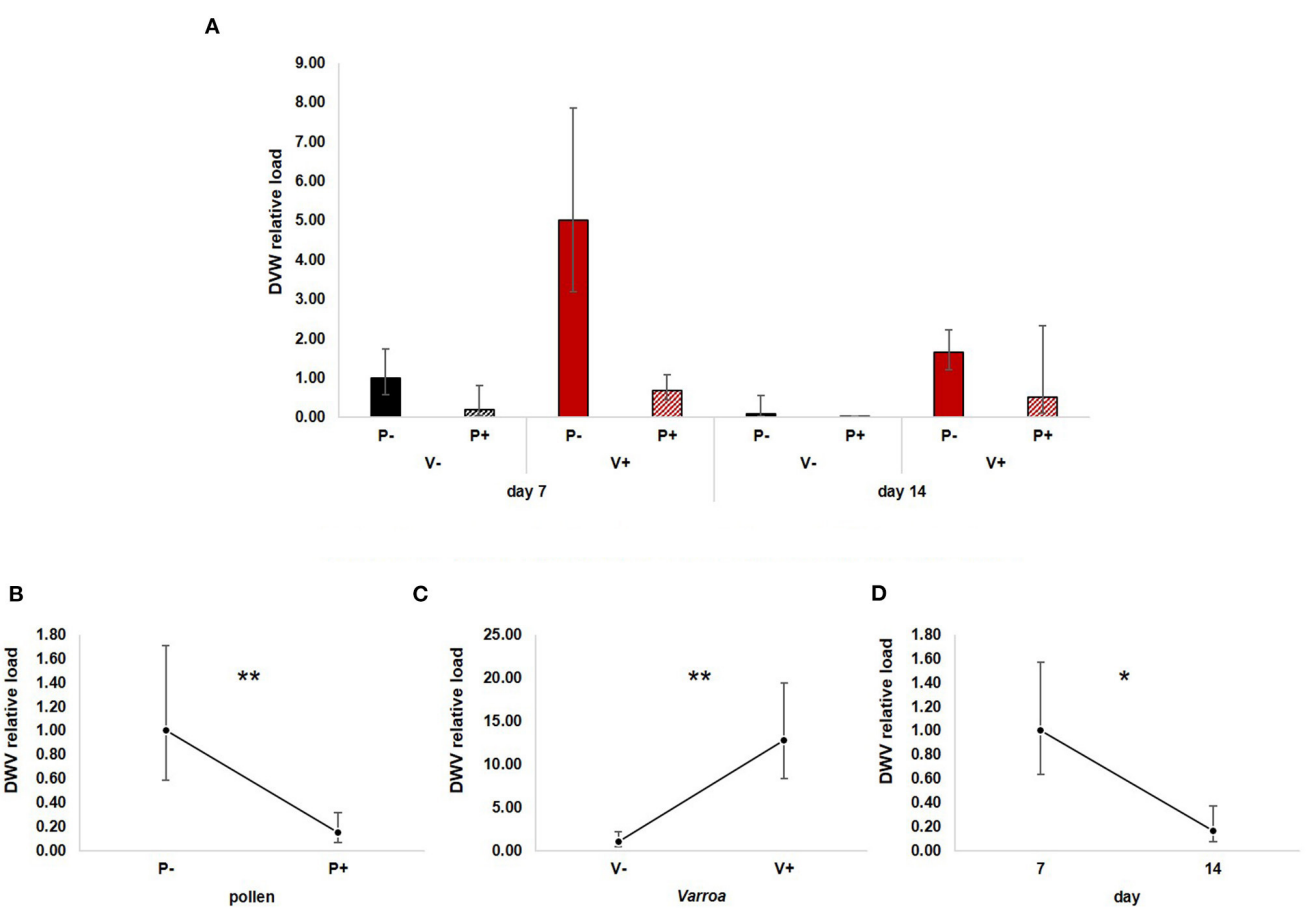
**(A)** DWV relative load in the experimental groups. **(B)** Pollen diet caused a significant reduction of viral load both in mite-infested and uninfested honey bees [GLM, pollen (*F* = 8.03, d.f. = 1, *P* = 0.007)]. **(C)** Varroa infestation increased virus load [Varroa (*F* = 12.94, d.f. = 1, *P* = 0.001)]. **(D)** DWV load decreased with time [GLM, time (*F* = 5.07, d.f. = 1, *P* = 0.029)]. *P < 0.05; **P < 0.01.

DWV is normally present in honey bees at low titers, but replication is activated by several stress factors, including Varroa infestation (19). Considering the decreasing immune-competence of foragers, we tested if the abundance of this ubiquitous pathogen is affected by age and how pollen feeding influences this interaction (Figure 6A).

GLM analysis revealed a significant negative effect of pollen on DWV load [Figure 6B; GLM, pollen (*F* = 8.03, d.f. = 1, *P* = 0.007)], a predictable increase of virus load modulated by Varroa infestation [Figure 6C; GLM, Varroa (*F* = 12.49, d.f. = 1, *P* = 0.001)] and a significant reduction with time [Figure 6D; GLM, time (*F* = 5.07, d.f. = 1, *P* = 0.029)]. No significant interactions between pollen, Varroa and time were noted (Supplementary Figures 5A–C).

## Discussion

Earlier studies demonstrated that dietary pollen has a positive effect on the immunity and lifespan of honey bees, while Varroa parasitization has a negative impact on these two traits. Our study identifies a potential mechanism for these pleiotropic effects. We demonstrate that pollen consumption alters the expression of two key genes underpinning the juvenile hormone-mediated behavioral maturation process in honey bee workers. Thus, pollen-fed bees are in a physiologically younger state, which is associated with increased immune function and hence increased ability to reduce viral infections. Since Varroa feeding shifts the expression of these key genes to accelerate maturation, parasitization can reduce longevity and immune gene function, leading to higher viral infections. Thus, dietary pollen can mitigate the impact of Varroa parasitization on bee immunity and lifespan, through its influence on these core genes.

In agreement with our previous study, the access to dietary pollen appeared to mitigate the impact of Varroa mite infestation in caged honey bees under laboratory conditions (38). Indeed, Varroa reduced the survival of honey bees, but pollen feeding nearly compensated for that effect, significantly extending the lifespan of mite-infested bees, such that the survival of pollen-fed mite-infested bees was not very different from that of pollen-fed uninfested bees. Indeed, apart from the first few days, when mite-infested bees fed with pollen survived less than uninfested bees, the survival curves of the two groups overlapped at around day 20 (Figure 1). These patterns are consistent with the results obtained for the DWV titers: at eclosion, mite-infested bees bear much higher levels of virus which tend to cause higher mortality in the very first days of adult life, before pollen consumption can exert its beneficial action.

Besides confirming previous results on the effects of pollen on the survival of mite-infested bees, in this work we wanted to test the hypothesis that pollen can prolong the lifespan of those bees by mitigating the accelerated behavioral maturation caused by the parasite. In particular, given the role played by Vitellogenin and JH on bees' behavioral maturation (36, 39), we first predicted that pollen stimulates the expression of *vg* and *jhe* in mite-infested bees. The role of pollen, influencing behavioral maturation via its effects on *vg* and *jhe*, was confirmed here. Both *vg* and *jhe* were up-regulated in pollen-fed bees (Figures 2B, 3B). The effect of pollen on Vitellogenin is consistent with results from previous studies (45). The effect of pollen on *jhe* levels, which is regarded as a marker of JH levels (35), supports the double repressor hypothesis proposed by Amdam and Omholt (36), where the transition to foraging is regulated by those two signals linked in a mutual negative feedback loop, generating the bistability responsible for the sharp transition between the two stages. Under this model, pollen can extend the lifespan of bees due to its action on these two regulators and the resulting delayed transition to foraging (43, 46). Furthermore, the accelerated behavioral maturation caused by the mite (27, 28, 47) was confirmed here by the down-regulation of *jhe* observed in the case of mite infestation (Figure 3C). While our study did not show a significant negative impact of *vg* levels as a result of mite infestation, possibly due to high variation among our samples, previous studies have clearly demonstrated that mite infestation results in a significant reduction of *vg* levels (28, 48, 49). Importantly, our results confirm our first prediction that the increase in the expression of *vg* [previously observed also by Alaux et al. (13)] and *jhe*, observed in mite-infested pollen-fed bees, can counteract the accelerated transition to foraging caused by mite infestation described above. Our results also support our second prediction, that pollen feeding stimulates immune function, as an outcome of delayed maturation. The fact that aging is related to a reduced immune competence is supported by the decreasing trend observed in both AMPs according to bees' age (Figures 4D, 5D). Moreover, the higher expression of both AMPs in pollen-fed bees indicates that indeed pollen feeding results in a delayed behavioral maturation and consequently a nurse-like phenotype at older ages (Figures 4B, 5B). In contrast, the up-regulation of AMPs in the case of mite infestation has already been observed (Figures 4C, 5C) (13, 28, 50, 51), and is likely related to the response to the secondary infections triggered by the mite (52), and the proposed implication of AMPs in antiviral response of bees (19, 53). Lastly, the results obtained here by testing DWV load in bees fed or not with pollen (Figure 6B), nicely confirm that, by postponing the transition to foraging and thus enhancing immune-competence, dietary pollen can indirectly contribute to reducing viral infections thus confirming our third prediction. The detrimental effects of *V. destructor* parasitism on DWV load have been extensively studied, and our results confirm previous data (13, 19, 38, 54, 55). Interestingly, there was a significant effect of time on DWV (Figure 6D). However, this result is mostly affected by the decreasing of virus load in sugar-fed bees from day 7 to day 14 (Supplementary Figure 5C). However, on day 14, more than 50% of the bees in this treatment group were already dead (Figure 1). Thus, the most infected bees in this sample group died early, likely leaving the less infected bees, which were sampled on day 14.

In conclusion, we confirm that pollen has a beneficial effect on bees challenged with Varroa mite. Varroa infestation at the pupal stage influences the nutritional status of the honey bee (23, 24); this compromises the natural maturation by influencing the relationship between two core physiological factors, Vitellogenin and JH (27). This leads to an accelerated transition to foraging and thus an anticipated death since this transition determines the overall lifespan of the bee (25). Instead, dietary access to pollen counteracts the accelerated transition caused by Varroa, influencing the key regulators of that process. As a further positive side effect, the enhanced immune-competence allows a better response to the secondary infections triggered by the mite, resulting in further reinforcement of the already positive effects of pollen on honey bee survival in case of mite infestation.

In our opinion, the lab work described here lays the foundations for further and necessary field-based studies. Our results well explain the effect of pollen on mite-infested individual bees but the complexity of social life could not be incorporated into our experiments. Indeed, the colony is supported by a complex network of interactions and the behavioral maturation of individual honey bees is affected by a number of external factors. In fact, in the colony, the transition to foraging is influenced by both social and environmental factors (56–59) but can also impact the colony's food intake as well as the individual bee mortality and thus colony composition and in turn pollen availability (56, 57). Furthermore, such colony effects can also influence Vitellogenin and JH levels (60). Further studies at colony level are therefore necessary to fully evaluate the effects of pollen on parasitized honey bees in their natural environment.

## Data Availability Statement

The original contributions presented in the study are included in the article/Supplementary Material, further inquiries can be directed to the corresponding author/s.

## Author Contributions

DF: conceptualization, data curation, investigation, and writing original draft. AR and CG: conceptualization. ES and VZ: data curation and investigation. DA: conceptualization, data curation, and investigation. FN: conceptualization, data curation, and writing original draft. All authors contributed to the article and approved the submitted version.

## Funding

This research was funded by the European Union's Horizon 2020 research and innovation programme, under Grant Agreement No. 773921 (PoshBee) and by the Italian Ministry of University, PRIN 2017 - UNICO (2017954WNT).

## Conflict of Interest

The authors declare that the research was conducted in the absence of any commercial or financial relationships that could be construed as a potential conflict of interest.

## Publisher's Note

All claims expressed in this article are solely those of the authors and do not necessarily represent those of their affiliated organizations, or those of the publisher, the editors and the reviewers. Any product that may be evaluated in this article, or claim that may be made by its manufacturer, is not guaranteed or endorsed by the publisher.
