# Supplementary material for: The Beneficial Effect of Pollen on Varroa Infested Bees Depends on Its Influence on Behavioral Maturation Genes
Source: Front Insect Sci. 2022 Apr 27;2:864238. doi: 10.3389/finsc.2022.864238 (PMC10926424; doi:10.3389/finsc.2022.864238)
Supplement: Supplementary file 2 [file Table_1.DOCX]

**The beneficial effect of pollen on *Varroa* infested bees depends on its effects on behavioural maturation genes**

Davide Frizzera, Allyson Ray, Elisa Seffin, Virginia Zanni, Desiderato Annoscia, Christina Grozinger, Francesco Nazzi

Table S1. List of primers used for virus and gene expression

| Primers | Sequence (5’-3’) | Bibliography |
| --- | --- | --- |
| b-actin F | CGTGCCGATAGTATTCTTG | (1) |
| b-actin R | CTTCGTCACCAACATAGG | (1) |
| GAPDH F | GCTGGTTTCATCGATGGTTT | (2) |
| GAPDH R | ACGATTTCGACCACCGTAAC | (2) |
| *vg* F | TTGACCAAGACAAGCGGAACT | (3) |
| *vg* R | AAGGTTCGAATTAACGATGAA | (3) |
| *jhe* F | GTTATCGCTTCTGATATGGCT | (4) |
| *jhe* R | GATGGGAAATAGGTACCGAC | (4) |
| *Apidecin-1* F | TTTTGCCTTAGCAATTCTTGTTG | (5) |
| *Apidecin-1* R | GAAGGTCGAGTAGGCGGATCT | (5) |
| *Defensin-1* F | CATGGCTAATGCCGGAGAGG | (5) |
| *Defensin-1* R | CTGCACCAGCTTGAAGAGC | (5) |
| DWV F | GGTAAGCGATGGTTGTTTG | (1) |
| DWV R | CCGTGAATATAGTGTGAGG | (1) |

**Bibliography**

1. Mondet F, de Miranda JR, Kretzschmar A, le Conte Y, Mercer AR. On the Front Line: Quantitative Virus Dynamics in Honeybee (*Apis mellifera* L.) Colonies along a New Expansion Front of the Parasite Varroa destructor. *PLoS Pathog*. (2014) 21;10(8):e1004323. doi: 10.1371/journal.ppat.1004323

2. Scharlaken B, de Graaf DC, Goossens K, Brunain M, Peelman LJ, Jacobs FJ. Reference Gene Selection for Insect Expression Studies Using Quantitative Real-Time PCR: The Head of the Honeybee, *Apis mellifera*, After a Bacterial Challenge. *J. Insect Sci*. (2008) 8(33):1–10. doi: 10.1673/031.008.3301

3. Fischer P, Grozinger CM. Pheromonal regulation of starvation resistance in honey bee workers (*Apis mellifera*). *Sci. Nat*. (2008) Aug 15;95(8):723–9.

4. Mackert A, do Nascimento AM, Bitondi MMG, Hartfelder K, Simões ZLP. Identification of a juvenile hormone esterase-like gene in the honey bee, *Apis mellifera* L. expression analysis and functional assays. *Comp. Biochem. Physiol. B. Biochem. Mol Biol*. (2008) 150(1):33–44. doi: 10.1016/j.cbpb.2008.01.004

5. Evans JD, Aronstein K, Chen YP, Hetru C, Imler J-L, Jiang H, et al. Immune pathways and defence mechanisms in honey bees *Apis mellifera*. *Insect Mol. Biol*. (2006) 15(5):645–56. doi: 10.1111/j.1365-2583.2006.00682.x
