# Supplementary figures and images for: The Beneficial Effect of Pollen on Varroa Infested Bees Depends on Its Influence on Behavioral Maturation Genes
Source: Front Insect Sci. 2022 Apr 27;2:864238. doi: 10.3389/finsc.2022.864238 (PMC10926424; doi:10.3389/finsc.2022.864238)

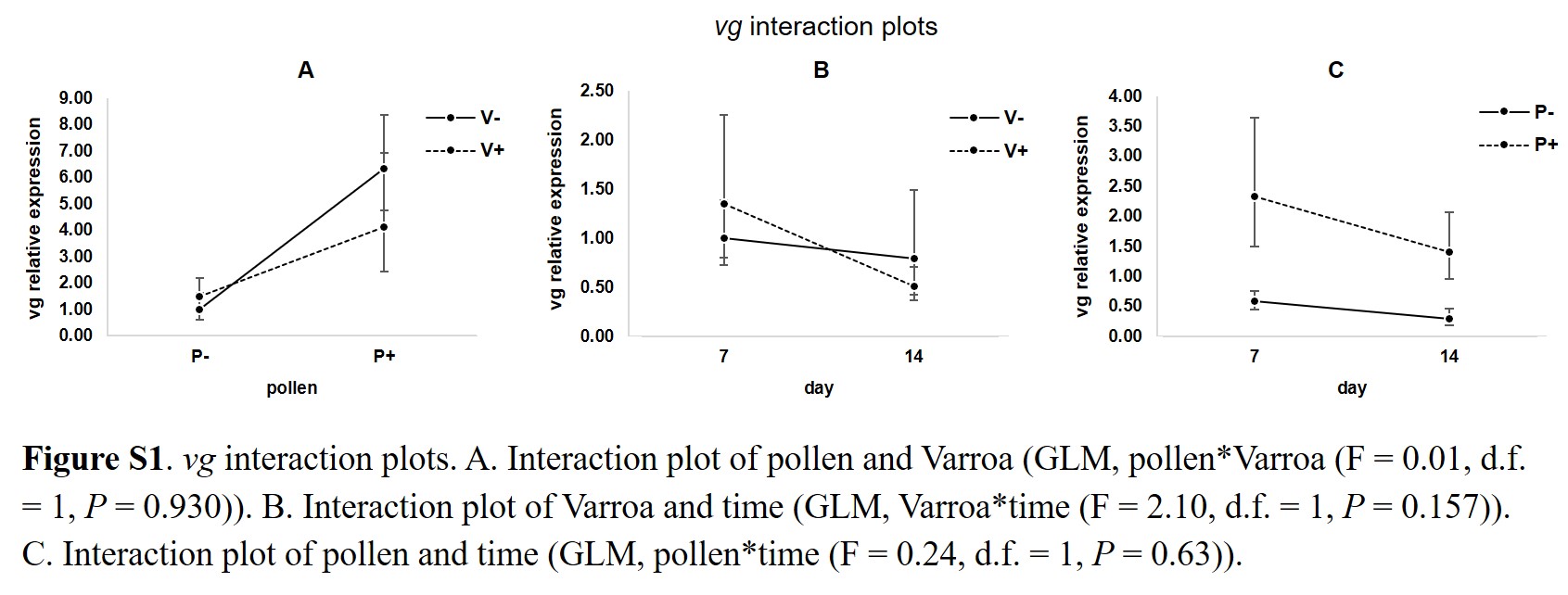

Supplement: Supplementary file 3 [file Image_1.JPEG]

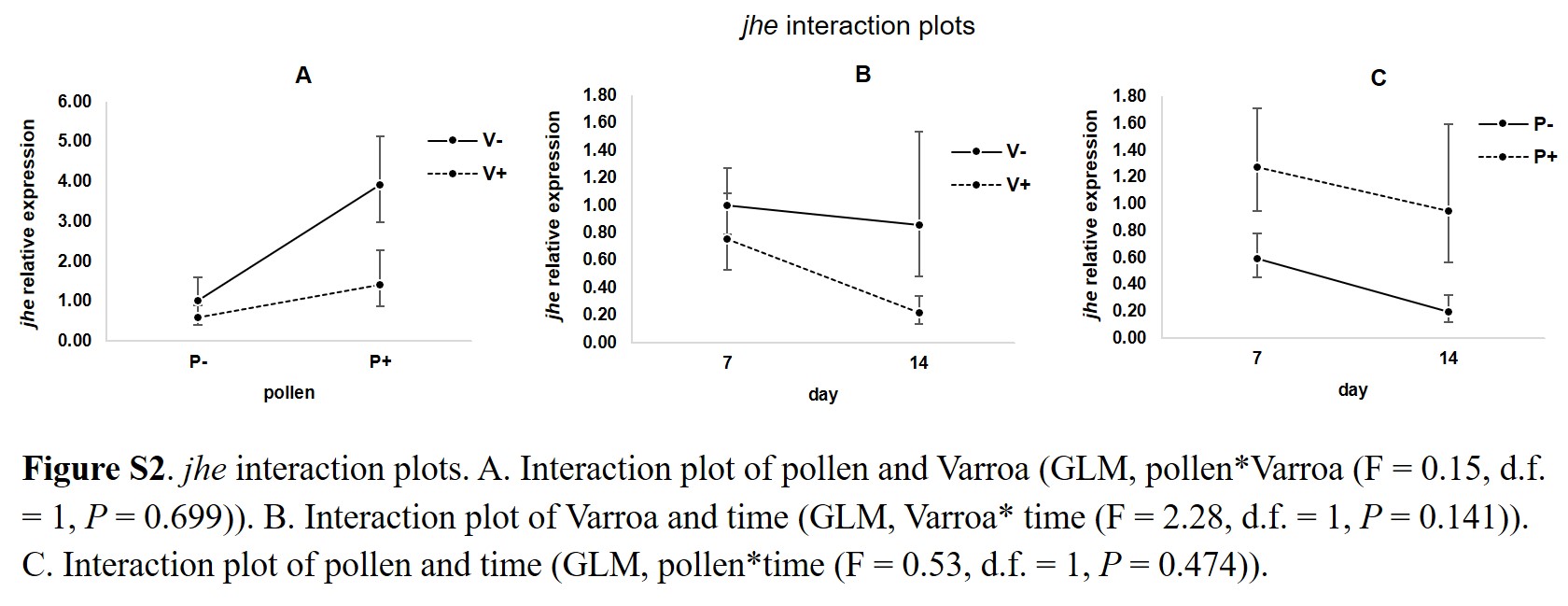

Supplement: Supplementary file 4 [file Image_2.JPEG]

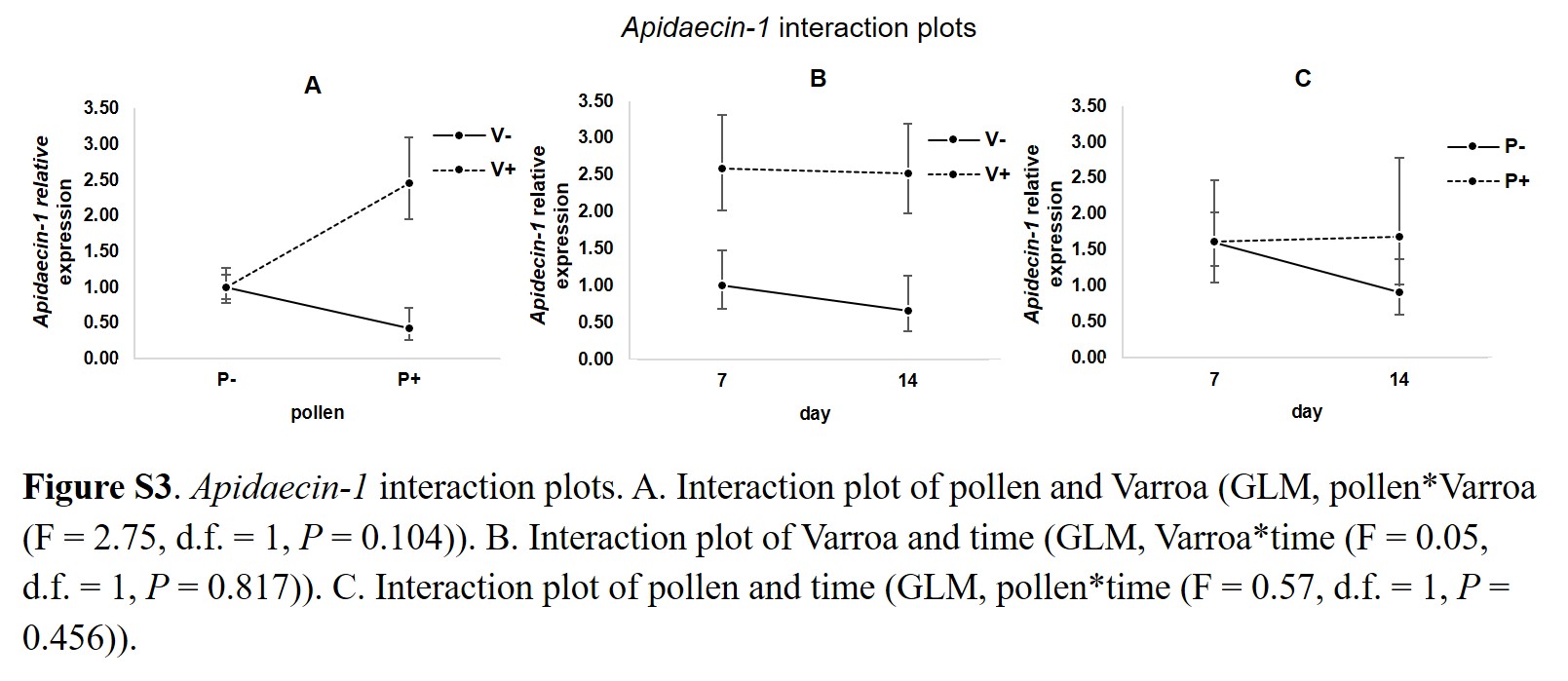

Supplement: Supplementary file 5 [file Image_3.JPEG]

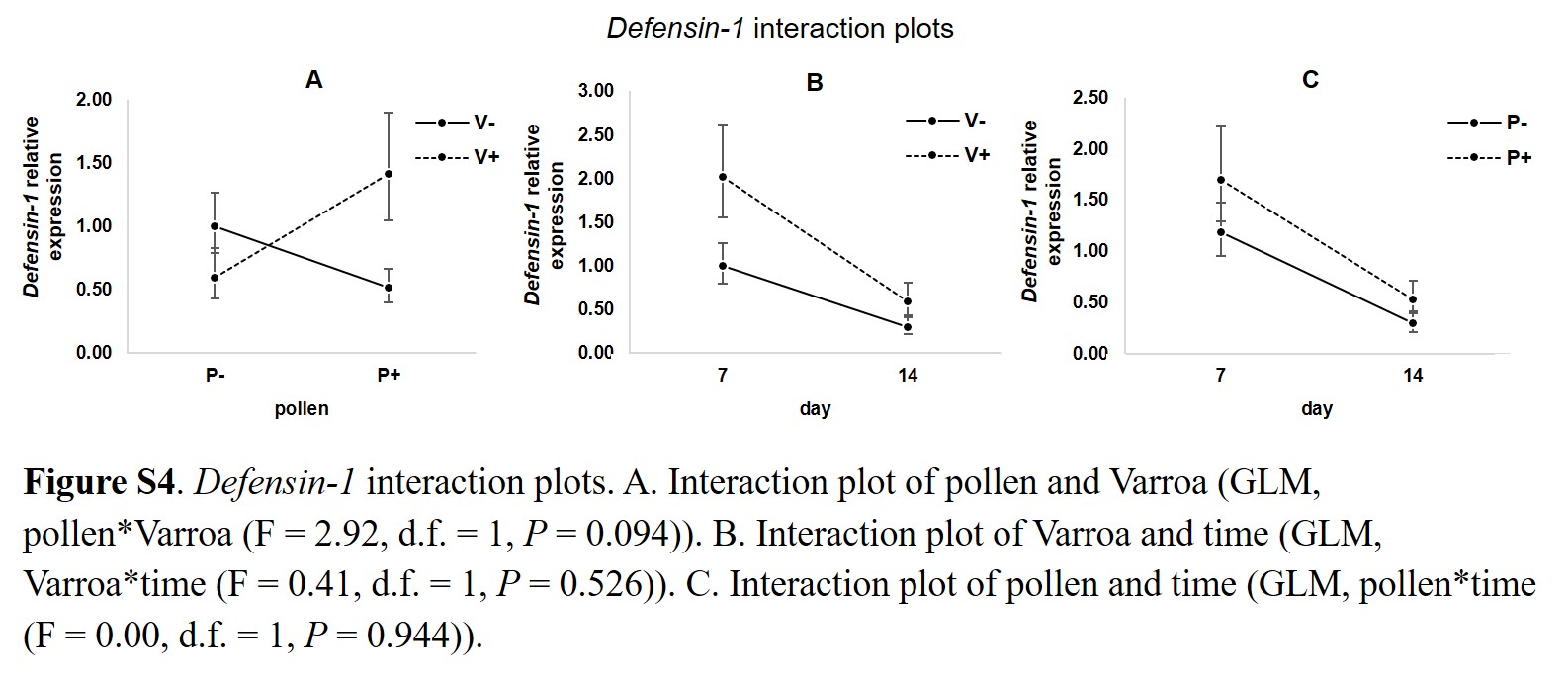

Supplement: Supplementary file 6 [file Image_4.JPEG]

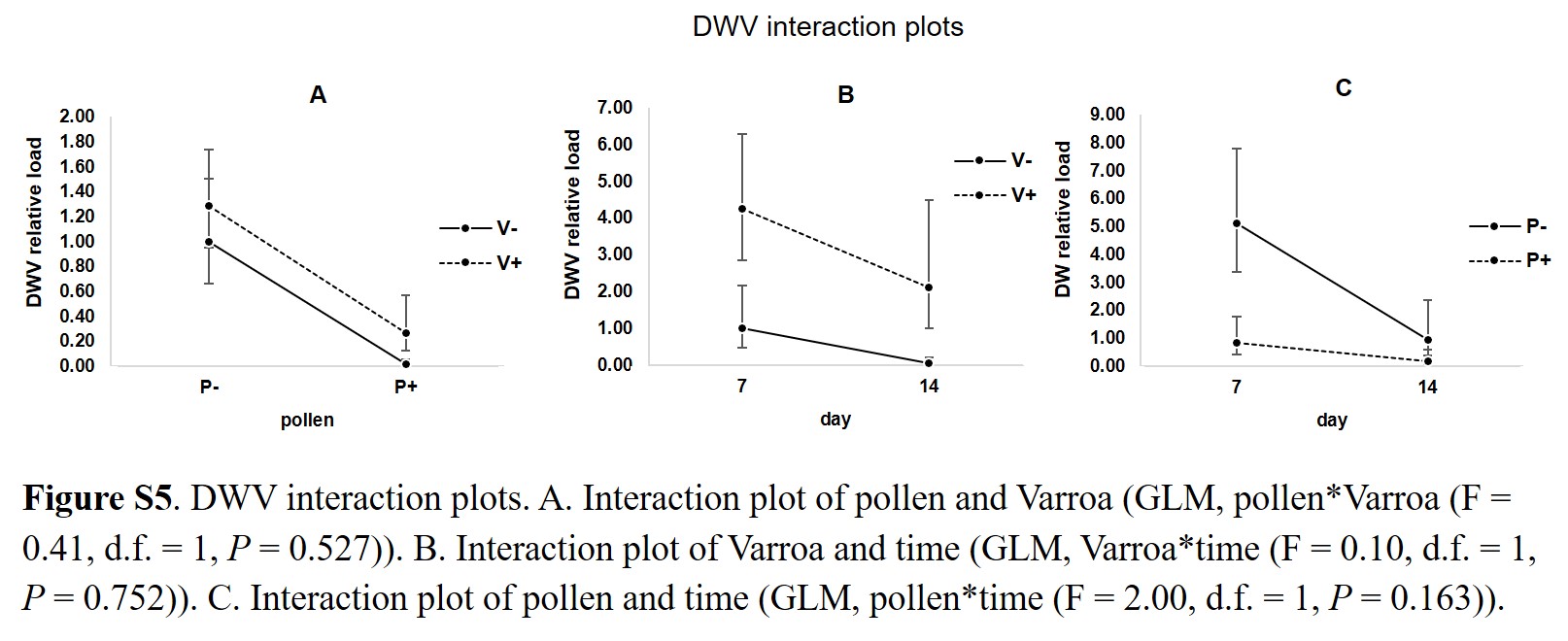

Supplement: Supplementary file 7 [file Image_5.JPEG]
